# Liver Abscess as an Uncommon Complication of Laparoscopic Appendectomy: A Case Report

**DOI:** 10.7759/cureus.54713

**Published:** 2024-02-22

**Authors:** David Savage-Lobeck, Nicholas Pereira, Robert Saggi

**Affiliations:** 1 Medicine, St. James School of Medicine, Chicago, USA; 2 Pediatrics, South Texas Health System Children’s, Edinburg, USA; 3 General Surgery, South Texas Health System Children's, Edinburg, USA

**Keywords:** pediatric hepatobiliary surgery, case report, appendicitis, appendectomy, pyogenic liver abscess

## Abstract

Pyogenic liver abscess (PLA) is an infrequently seen complication of appendicitis that once was common, but now is so rare many textbooks omit the condition entirely. In this report, we document a recent case of post-appendicitis PLA in an eight-year-old Hispanic female treated with a combination of medical and surgical management. We have detailed the course of treatment, both to raise awareness of this uncommon complication of appendicitis and to help provide a guide for other clinicians treating similar cases. While cases of pediatric PLA post-appendicitis are rare in the modern world, timely diagnosis and treatment of the lesions are paramount to patient recovery and prevention of long-term sequelae. Study of prior literature and research is likely to be of vital importance to the treatment of the condition. Multiple treatment modalities may be considered, and there is no true standard of care for pediatric populations presenting with PLA.

## Introduction

Dieulafoy originally described pyogenic liver abscess (PLA) as a possible complication of appendicitis in 1898 [1,2]. Since the rise of antibiotics, the incidence of PLA in this context has decreased markedly. Prior to this, however, appendicitis was a common cause of PLA. In 1938, Oschner and Debakey noted appendicitis to be the most likely root cause of 11%-34% of cases of PLA [2]. The study also described the overall incidence of PLA after appendicitis at the time, which was estimated at 0.1%-0.4% [2]. With improvements in treatment methods and surgical techniques, appendicitis presenting as a direct cause of PLA has been reduced from the prior listed values to 3%, with PLA not presenting at even 0.1% of appendicitis [3]. This complication is rarely documented in the United States; however, Thavamani et al. estimated the modern incidence of pyogenic liver abscess in the United States was 25 per 100,000 children, using the National Inpatient Sample and Kids Inpatient databases. Of those children, 61% of affected children were boys, and the mean age was 13.03 ± 6.1 years [4]. The main documented comorbid conditions associated with PLA were hepatobiliary malignancy (odds ratio [OR] = 71.8) followed by liver transplant (OR = 38.4), and biliary disease (OR = 29.9). Appendicitis was also listed, albeit with a less severe correlation, with an OR of 1.8 [4]. 

## Case presentation

**Figure 1:**
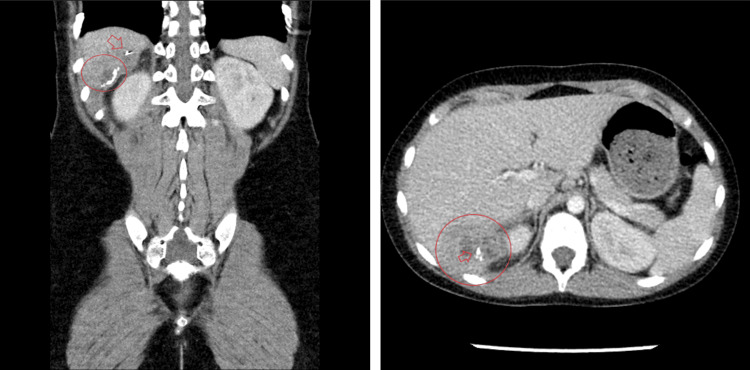
Computed tomography images showing a hepatic abscess taken one day before admission. The coronal and axial views shown display clearly the lesion in question (circled region), with early evidence of calcifications (radiolucent areas marked with arrows). In the coronal view, an area of aberrant calcification is marked. The red circle shown on the axial view shows inflammation of the kidney and liver; the heterogeneous lesion is clearly noted in the liver, and locally extends medially to the kidney.

An eight-year-old Hispanic female presented to a referring hospital with fever two weeks after an uncomplicated laparoscopic appendectomy for gangrenous appendicitis. One day before presenting to the hospital, the patient visited an outpatient computed tomography (CT) center, where a scan of the abdomen showed a 4.3 cm x 4.5 cm x 3 cm heterogeneous lesion in the right lower lobe of the liver; images of these scans are shown in Figure 1. The imaging also showed inflammation of the right kidney, compatible with an abscess extending to this area. An enlarged internal right lower abdominal lymph node was seen with a size of 1.5 cm. No thrombosis of the portal vein was seen. Based on these results, the patient was referred to the hospital. 

**Figure 2:**
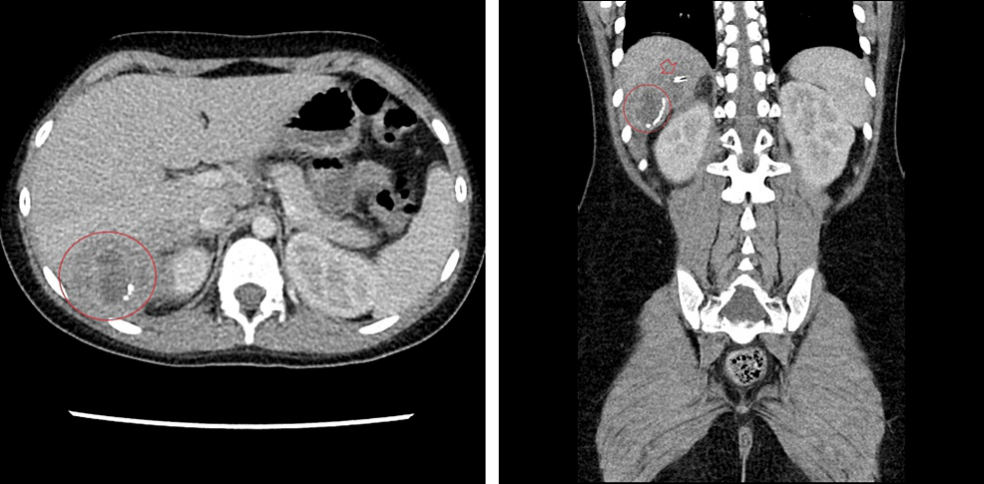
Computed tomography image showing a hepatic abscess taken on the third day of admission. Along the inferomedial border of the lesion in the coronal view, increasing levels of calcifications can be seen forming a radiolucent border. Superomedial to the lesion, an area of radiolucency is seen (arrow); while previously ambiguous on imaging, it appears to be clip-like in shape, but clinical history makes this unlikely.

On the day of admission, the patient was febrile to 38°C. Peripheral blood leukocytosis was present at 14,400 cells/high power field (hpf) (normal range adjusted for age, 6200-8550 cells/hpf). Other vitals and labs were stable and within normal limits. She appeared well, with no complaints of pain. Physical exam showed a soft abdomen without guarding or rigidity. She was admitted to medicine, and was started on piperacillin-tazobactam. Over the next 12 hours, she developed bilious vomiting, and metronidazole was begun on continuous administration for additional anaerobic coverage since the identity and resistance of the offending microbe were not yet known. At this time, the hospital’s infectious disease and general surgery teams recommended drainage of the lesion, instead of continuing medical treatment alone. The patient was also started on a clear-fluid diet. Blood cultures showed no growth by the third day of admission, and a repeat CT showed progression of the abscess to 5.5 cm x 4.7 cm x 5.2 cm, with increased levels of organization throughout the lesion. MRI and ultrasound were considered to conserve radiation exposure to the patient, but were ruled out based on MRI availability and the concomitant desire for complete abdominal cavity visualization. The previously documented calcifications were noted to have enlarged from their previous size during the procedure, with the most superior of these being suggestive of either a slipped surgical clip or appendicoliths as possible focus for the abscess; however, during an interview with the surgical team, it was revealed that the appendectomy had been done with staples, not clips, making an appendicolith a more likely source, given the patient’s lack of other significant surgical history. These images are shown in Figure 2.

On the fourth day of admission, CT-guided percutaneous drainage was performed. At this time, the lesion measured 5.5 cm x 4.7 cm x 5.2 cm. A 6 French pigtail drain was placed for continuous drainage. 5mL of purulent fluid was aspirated from the lesion, and 10mL more was drawn from the catheter after the procedure.

Post-operatively, the patient experienced a slight fever which subsided until the next morning, when she had another. This subsided once more as the day progressed. Other vitals remained stable and within normal limits. She transitioned to soft-fiber foods, then a full diet later in the afternoon without issue. Wound culture grew colonies of extended-spectrum beta-lactamase-producing Escherichia coli, as well as a few gram-positive organisms suspected to be gut flora. Susceptibility testing revealed that the strain in question was non-resistant to piperacillin-tazobactam, so treatment adjustment was not required at this time. Over the next three post-drainage days, the patient had several febrile episodes, ranging from 37.2°C to 39.4°C. Due to resistant fever, on the third post-drainage day, piperacillin-tazobactam was discontinued and meropenem was started at a rate of 20 mg/kg every eight hours. Further imaging modalities with non-ionizing radiation were discussed, and a consensus was reached that ultrasound would be a preferable option to further CT scans.

**Figure 3:**
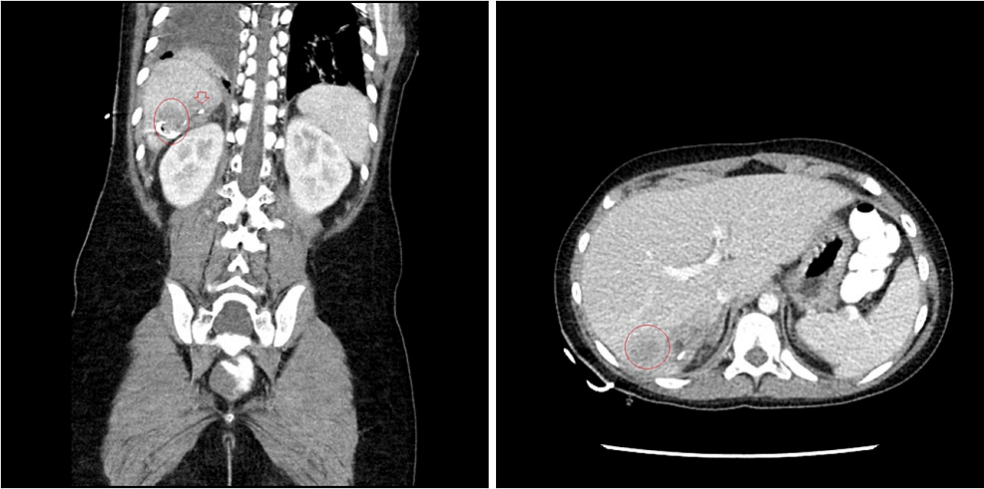
Computed tomography image showing a hepatic abscess taken on the fifth day post-drainage Along the inferior border of the abscess cavity, laying along previously noted calcifications, coiling excess drain tube can be seen as a slightly spiraled area of radiolucency within the red circle shown in the coronal view. Compared to prior images, the abscess volume is greatly reduced. The red arrow points to the previously observed area of radiolucency, thought to be a clip, which had remained in place superomedial to the main lesion throughout admission at the time of imaging.

An ultrasound was performed on the fourth day post-drainage, showing a 2.8 cm x 4.3 cm x 3.1 cm heterogeneous area in the position of the healing abscess. Biliary dilation was absent, and portal venous flow was noted to be normal. Radiologic interpretation of a CT scan on postoperative day five showed a 3 cm x 5.3 cm x 2.8 cm heterogeneous area of enhancement/attenuation; these images are shown in Figure 3. The previously noted object considered to be a clip or appendicolith was still in place, and on the 10th day of admittance an exploratory laparotomy was performed due to continued fever. The laparotomy showed no significant findings, and further treatment was limited to IV antibiotics and symptomatic management. The patient was discharged on the 20th day of admittance to the hospital once the fever subsided.

Outpatient CT imaging on the 31st day after admittance showed the liver lesion decreasing significantly in size since prior studies, with the drainage system still in place. Outpatient follow-ups without additional imaging with the patient's family doctor at the one-month and three-month marks of discharge reported no further issues involving the lesion. 

## Discussion

Due to the inherent rarity of PLA as a post-appendectomy complication in pediatric patients, the default management of these patients usually follows the guidelines for adult patients, which call for guided drainage of lesions less than 30-50 mm. Ayers et al. described the case of PLA in an eight-year-old male, presenting with similar characteristics to the patient in our case [5]. However, their patient was managed with antibiotics, and showed little improvement, both in disease course or imaging, over the course of a month. After surgical drainage of the lesion, the patient improved significantly; he transitioned to a stable afebrile state at home on IV antibiotics and remained asymptomatic. Imaging after drainage showed the lesion healing well [5]. This correlation suggests early drainage of even a small lesion may be a viable, and potentially first-line, treatment option for PLA in pediatric patients, in contrast to medical management alone. In the Ayers study, the use of several smaller-spectrum antibiotics (notably amoxicillin-clavulanate and piperacillin-tazobactam) were tried for several weeks before drainage was attempted; in our case, a stronger antibiotic (meropenem) as well as concurrent drainage were implemented to treat the abscess [4]. Future studies should be analyzed comparing the methods to help shape treatment guidelines for pediatric patients. 

Medical management of PLA in pediatrics without surgical intervention has been the default treatment modality, especially with smaller lesions. Kumar et al. treated 66% of cases of PLA seen in a South Indian hospital over a six-year period with only antibiotics; however, 16.7% of those cases still required drainage [6]. Another report of 38 children in Pakistan with PLA showed a good response to medical treatments in smaller abscesses (<5 mm), but also revealed better outcomes with percutaneous drainage in larger lesions (>5 mm) [7]. Other case reports reinforce the importance of surgical intervention by demonstrating worse outcomes with medical management alone [3,8,9]. Typical microbes associated with PLA, especially in pediatric patients, tend to belong to the Streptococcus (10.8%) and Staphylococcus spp (9.2%) [4]. In worldwide populations, Staphylococcus aureus is the most common pathogen involved in the development of PLA [10].

Prior cases of pediatric PLA have not only been shown to have decreased medical responsiveness but have also indicated better response to surgical management than medical interventions. Ayers et al. is an excellent example of surgical intervention displaying superiority in illness resolution in the case of PLA [4]. Mishra et al. described percutaneous drainage (especially of unilocular lesions) as the treatment modality of choice for PLA in pediatric populations in several situations, notably if there is failure of medical treatment after 48-72 hours of antibiotic administration. This suggestion was associated with positive outcomes in both the Ayers et al. case and our own. Further indications for drainage under their study included impending rupture (notably PLA on the left side of the liver), a large abscess area, evidence of liver failure, and as a temporal measure pre-surgically to improve conditions [10]. Further studies indicate that a total of 80-90% of PLA managed medically may require eventual drainage [11,12]. Huang et al. described the success of concurrent antibiotic treatment and percutaneous drainage in adults in treating PLA, but it stands unknown if this translates into pediatric populations well [12]. 

In regard to surgical management of PLA, there is also some debate remaining on the effectiveness of percutaneous vs open drainage being a superior treatment modality. It is recommended here that thought be given to both methods, tailoring treatment to each case’s etiology and characteristics until more conclusive research can be completed into the advantages and drawbacks of both techniques [13-16]. It is thought that individual skill plays a role in the effectiveness of percutaneous drainage [13], so it stands to reason that as imaging modalities improve and skills develop, the incidence and success of percutaneous drainage will increase with time. Kajala et al. described USG drainage with placement of a drainage catheter to be superior to drainage alone, especially with larger abscesses [17].

## Conclusions

We have chosen to describe this pediatric case of PLA as a complication of appendectomy to not only reveal the importance of considering PLA in differential diagnoses as a cause of fever and vague infectious symptoms in a child post-appendectomy (even after an extended period of time) but also display the vital role drainage plays in the treatment of PLA in pediatric patients, as well as discuss the multivariate treatment approaches the condition inherently invites.
